# Proteomics data for characterizing *Microbacterium oleivorans A9*, an uranium-tolerant actinobacterium isolated near the Chernobyl nuclear power plant

**DOI:** 10.1016/j.dib.2018.10.136

**Published:** 2018-10-30

**Authors:** Nicolas Gallois, Laurie Piette, Philippe Ortet, Mohamed Barakat, Justine Long, Catherine Berthomieu, Jean Armengaud, Virginie Chapon, Béatrice Alpha-Bazin

**Affiliations:** a CEA, CNRS, Aix-Marseille Université, UMR 7265 Biologie Végétale et Microbiologie Environnementales, Laboratoire des Interactions Protéine Métal, 13108 Saint-Paul-lez-Durance, France; b CEA, DRF/Joliot/SPI/Li2D, BP 17171, F-30200 Bagnols-sur-Cèze, France; c CEA, CNRS, Aix-Marseille Université, UMR 7265 Biologie Végétale et Microbiologie Environnementales, Laboratoire d׳écologie microbienne de la rhizosphère et d׳environnements extrêmes, 13108 Saint-Paul-lez-Durance, France

## Abstract

*Microbacterium oleivorans* A9 cells were exposed or not to 10 µM uranyl nitrate as resting cells in sodium chloride solution. Bacteria exposed to U(VI) and controls were harvested after 0.5, 4, and 24 h of toxicant exposure. Bacteria were subjected to high-throughput proteomics analysis using a Q-Exactive HF high resolution tandem mass spectrometer incorporating an ultra-high-field orbitrap analyzer. MS/MS spectra were assigned with a protein sequence database derived from a draft genome obtained by Illumina sequencing and systematic six-reading frame translation of all the contigs. Proteins identified in bacteria exposed to U(VI) and controls at the three time points allow defining the proteome dynamics upon uranium stress. The data reported here are related to a published study regarding the proteome dynamics of *M. oleivorans* A9 upon uranium stress by Gallois et al. (in press) entitled “Proteogenomic insights into uranium tolerance of a Chernobyl׳s Microbacterium bacterial isolate”. The data accompanying the manuscript describing the database searches and comparative analysis have been deposited to the ProteomeXchange with identifier PXD005794.

**Specifications table**

**Table:** 

Subject area	*Environmental microbiology*
More specific subject area	*Actinobacteria comparative proteogenomics*
Type of data	*Figure, mass spectrometry raw files, Excel tables*
How data was acquired	*Data-dependent acquisition of tandem mass spectra using a Q-Exactive HF tandem mass spectrometer (Thermo).*
Data format	*Raw and processed*
Experimental factors	*Cells, at the exponential growth phase, were harvested and the resulting cell pellets were resuspended in NaCl solution with 0 (control) or 10 µM uranyl nitrate. For each of the three sampling time points, 0.5, 4 and 24 h, four biological replicates were performed.*
Experimental features	*The 24 proteomes were briefly run on SDS-PAGE, followed by trypsin proteolysis. Tryptic peptides were analyzed by nanoLC-MS/MS and spectra were assigned with a draft genome-derived protein sequence database.*
Data source location	CEA-Marcoule, DRF-Li2D, Laboratory “Innovative technologies for Detection and Diagnostics”, BP 17171, F-30200 Bagnols-sur-Cèze, France
Data accessibility	Data are within this article and deposited to the ProteomeXchange via the PRIDE repository with identifier PRIDE: PXD005794.

**Value of the data**

• The data are an interesting resource regarding the proteome content of a soil bacterium with extreme tolerance to heavy metals.
• The proteogenomics strategy described here allows a quick identification of proteins based on a draft genome of this high GC content organism.
• The data have been used to define the proteome changes in response to uranium stress in an Actinobacteria isolate from the trench T22 located near the Chernobyl nuclear power plant. As described in detail in the accompanying manuscript [1], the uranyl stress perturbed the phosphate and iron metabolic pathways.

## 1 Data

This report contains the complete list of peptide-to-spectrum assignments for the control (not treated) samples and Uranium-treated samples of *Microbacterium oleivorans* A9 in the first round of the proteogenomics cascade search (Supplementary Table S1), in the second round of the cascade search (Supplementary Table S2). A total of 746,092 MS/MS spectra were assigned in the first search round and 747,621 MS/MS spectra were interpreted in the second search round. All the tandem mass spectrometry characteristics (measured mass, charge, protease cleavage, post-translational modifications, and retention time) are indicated.

**Fig. 1:**
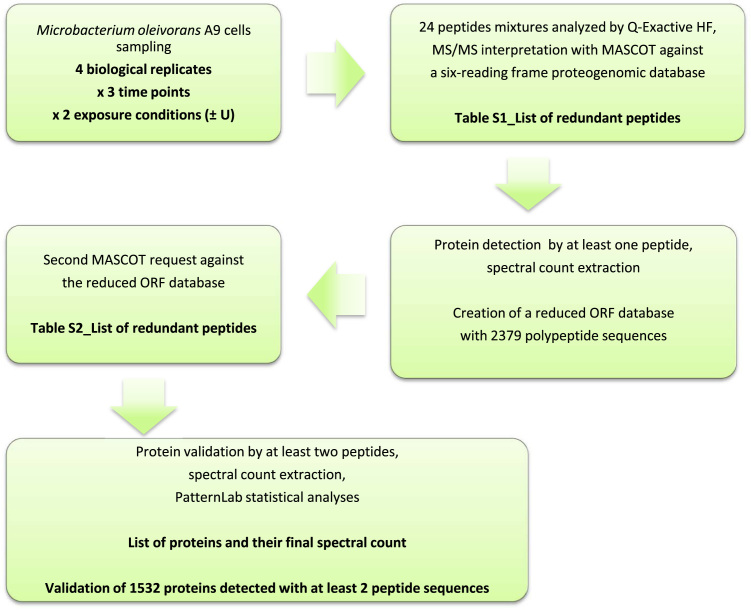
Flowchart of experiments, data processing and refined data tables.

Fig. 1 shows the schematic flowchart of experiments, data processing and results that were obtained and formatted in.xls tables. The proteome data from four independent biological replicates per time point (0.5, 4 and 24 h) upon uranium exposure or control, i.e. from 24 samples, were assigned to tryptic peptides against the *M. oleivorans* A9 ORF database described by Gallois *et al.* [1] following a proteogenomic approach [2], [3], [4]. The deposited data comprised the 24 raw files and the interpreted files. Supplementary Tables S1 and S2 list the peptide-to-spectrum matches with all the tandem mass spectrometry characteristics in the first query round against the large proteogenomics database and second query round against the reduced ORF database, respectively.

## 2 Experimental design, materials and methods

### 2.1 Preparation of *Microbacterium oleivorans* A9 samples

*M. oleivorans* A9 cells were isolated from Chernobyl trench T22 soil [5] and exposed as described [1]. Briefly, cells were harvested and the cell pellets were resuspended in 0.1 M NaCl pH 5.0 with 0 (control) or 10 µM uranyl nitrate. Four independent biological replicates were performed for statistical purpose. Fractions of 1 ml of cell suspension were taken after 0.5, 4, and 24 h for each condition. After centrifugation, the resulting supernatants were removed and the pellets were conserved at -80 °C until proteomic analysis. Proteogenomics were carried out as previously described [1], [6] taking into account the recent draft genome of the strain.

### 2.2 Protein extracts and tandem mass spectrometry

The 24 peptide mixtures were performed using a Q-Exactive HF mass spectrometer (ThermoFisher) coupled to an UltiMate 3000 LC system (Dionex-LC Packings) in similar conditions as those previously described. Peptide mixtures (10 μl) were loaded and desalted on-line on a reverse phase precolumn (Acclaim PepMap 100 C18) from LC Packings. Peptides were then resolved onto a reverse phase Acclaim PepMap 100 C18 column and injected into the Q-Exactive HF mass spectrometer. The Q-Exactive HF instrument was operated according to a Top20 data-dependent acquisition method as previously described [7].

### 2.3 Protein sequence database for proteogenomic MS/MS assignment

The recorded MS/MS spectra for the 24 samples were searched against our home-made ORF database with the parameters described previously [1]. This database contains 30,853 polypeptide sequences, for a total of 4,903,573 amino acids with an average of 159 amino acids per polypeptide. The number of MS/MS spectra per protein (spectral counts) was determined for the four replicates of each of the three time points for both conditions. The statistical protein variation was compared for each time point between the uranyl exposure and the control conditions using the T-Fold option of PatternLab 2.0 software [8].

## References

[1] Gallois N., Alpha-Bazin B., Ortet P., Barakat M., Piette L., Long J., Berthomieu C., Armengaud J., Chapon V. (2018). Proteogenomic insights into uranium tolerance of a Chernobyl׳s Microbacterium bacterial isolate. J. Proteom..

[2] Hartmann E.M., Armengaud J. (2014). N-terminomics and proteogenomics, getting off to a good start. Proteomics.

[3] Armengaud J., Hartmann E.M., Bland C. (2013). Proteogenomics for environmental microbiology. Proteomics.

[4] Armengaud J., Trapp J., Pible O., Geffard O., Chaumot A., Hartmann E.M. (2014). Non-model organisms, a species endangered by proteogenomics. J. Proteom..

[5] Chapon V., Piette L., Vesvres M.-H., Coppin F., Marrec C.L., Christen R., Theodorakopoulos N., Février L., Levchuk S., Martin-Garin A., Berthomieu C., Sergeant C. (2012). Microbial diversity in contaminated soils along the T22 trench of the Chernobyl experimental platform. Appl. Geochem..

[6] Hartmann E.M., Allain F., Gaillard J.C., Pible O., Armengaud J. (2014). Taking the shortcut for high-throughput shotgun proteomic analysis of bacteria. Methods Mol. Biol..

[7] Klein G., Mathe C., Biola-Clier M., Devineau S., Drouineau E., Hatem E., Marichal L., Alonso B., Gaillard J.C., Lagniel G., Armengaud J., Carriere M., Chedin S., Boulard Y., Pin S., Renault J.P., Aude J.C., Labarre J. (2016). RNA-binding proteins are a major target of silica nanoparticles in cell extracts. Nanotoxicology.

[8] Carvalho P.C., Hewel J., Barbosa V.C., Yates J.R. (2008). Identifying differences in protein expression levels by spectral counting and feature selection. Genet. Mol. Res..

